# CO_2_ Adsorption on Activated Carbons Prepared from Molasses: A Comparison of Two and Three Parametric Models

**DOI:** 10.3390/ma14237458

**Published:** 2021-12-05

**Authors:** Karolina Kiełbasa, Adrianna Kamińska, Oliwier Niedoba, Beata Michalkiewicz

**Affiliations:** Department of Catalytic and Sorbent Materials Engineering, Faculty of Chemical Technology and Engineering, West Pomeranian University of Technology in Szczecin, Piastów Ave. 42, 71-065 Szczecin, Poland; kaminska.adrianna@zut.edu.pl (A.K.); niedoba.oliwier@zut.edu.pl (O.N.); Beata.Michalkiewicz@zut.edu.pl (B.M.)

**Keywords:** activated carbon, CO_2_ adsorption, adsorption models, error analysis

## Abstract

Activated carbons with different textural characteristic were derived by the chemical activation of raw beet molasses with solid KOH, while the activation temperature was changed in the range 650 °C to 800 °C. The adsorption of CO_2_ on activated carbons was investigated. Langmuir, Freundlich, Sips, Toth, Unilan, Fritz-Schlunder, Radke-Prausnitz, Temkin-Pyzhev, Dubinin-Radushkevich, and Jovanovich equations were selected to fit the experimental data of CO_2_ adsorption. An error analysis (the sum of the squares of errors, the hybrid fractional error function, the average relative error, the Marquardt’s percent standard deviation, and the sum of the absolute errors) was conducted to examine the effect of using various error standards for the isotherm model parameter calculation. The best fit was observed to the Radke-Prausnitz model.

## 1. Introduction

In recent years, global warming has become a very serious problem. The global average temperature increased every year by around 1 °C because of the increase of greenhouse gas concentrations. Carbon dioxide plays the most crucial role in the greenhouse effect, as it remains much longer in the atmosphere than other gasses. The CO_2_ emissions increased from 2 billion tons in 1900 to over 36 billion tons in 2020 [1].

Since the steady growth of anthropogenic CO_2_ in the atmosphere is observed it is vital to engage in an ongoing effort to reduce the consequence of global greenhouse emissions causing climate change by establishing an effective approach for capturing CO_2_.

The application of porous materials for CO_2_ capturing is a promising strategy. Especially carbonaceous materials offer advantages of high stability, rapid kinetics, low desorption temperature. The cost of carbonaceous materials is very low if the raw materials are renewable sources or even waste. Therefore, many researchers are focused on developing technologies, leading to the capture and storage of carbon dioxide, especially adsorption techniques, which are currently considered very promising [2]. Recently, many solid adsorbents have been reported, which could be applied in the CO_2_ capture: activated carbons [3,4], zeolites [5], metalloorganic structures [6], porous polymers [7], carbon nanosheets [8], metal oxides [9], TiO_2_ modified by nitrogen [10], activated carbons-TiO_2_ composites [11], TiO_2_ modified by amines [12], TiO_2_/titanate composite nanorods [13], TEPA-modified titanate composite nanorods [14], carbon nanotubes [15], monoliths [16]. Among the above-mentioned materials, the carbonaceous materials are considered as particularly promising with regard to a low production cost, well developed porosity, large surface area, readily controlled structure, good thermal and chemical stability and large efficiency as well as the wide spectrum of usage [17,18].

The adsorption capacity and the efficiency of the activated carbon adsorption system are predicted from equilibrium sorption isotherms. The adsorption of gases and solutes is usually described through isotherms, that describing the relationship between an amount of adsorbed adsorbate and its equilibrium concentration in bulk solution at a constant temperature. The isotherm is used to characterize and evaluate the most important properties of adsorbent such as adsorbent affinity, adsorption capacity, adsorption mechanism and quantitative distribution of adsorbate on adsorbent and bulk solution. The adsorption process is described by isotherm models of two, three, four, and even five parameters [15,19,20]. To determine adsorption isotherm and its constant, experimental and calculation stages are required. Amongst the existing theoretical adsorption models Langmuir, Freundlich, Sips, Toth, Unilan, Fritz-Schlunder, Radke-Prausnitz, Temkin-Pyzhev, Dubinin-Radushkevich, and Jovanovich equations were selected to quantitatively compare the applicability of isotherm models for fitting the experimental data of the CO_2_ adsorption. The equations which define the absolute amount of adsorbed gas dependent on the pressure were described below:

### 1.1. Langmuir Isotherm

The Langmuir isotherm, which is the simplest model, was designed to characterize the adsorption of the gas-solid phase. It is also used to quantify and compare the maximum adsorption capacity of different sorbents. The Langmuir theory postulates monolayer coverage of adsorbate; adsorption occurs at specific homogeneous sites (all sites are equal, resulting in equal adsorption energies). Once an adsorbate molecule occupies a site, no more adsorption can take place at that site. The sorbent has a limited capacity for the adsorbate [21,22]. The Langmuir isotherm is expressed following Equation (1):

(1) $$q=\frac{q_{\mathrm{mL}}b_{L}p}{1+b_{L}p}$$

where q_mL_ is the maximum adsorption capacity [mmol/g], b_L_ is the Langmuir constant [bar^−1^], p is pressure [bar], q is the adsorbed quantity under p pressure [mmol/g].

### 1.2. Freundlich Isotherm

The Freundlich model describes an adsorption on heterogeneous surfaces with different adsorption energies [23,24] according to Equation (2):

(2) $$q=k_{F}{p\;}^{n_{F}}$$

where k_F_ is the Freundlich constant [mmol/g], n_F_ is the heterogeneity factor.

### 1.3. Toth Isotherm

The Toth isotherm model is the next empirical equation developed to enhance isotherm fitting between experimental and calculated data. The Toth isotherm model is useful in describing heterogeneous adsorption systems, which settles with both low and high-end boundaries of the concentration [25,26] according to Equation (3):

(3) $$\;q=\frac{q_{\mathrm{mT}}b_{T}p}{{\left(1+{\left(b_{T}p\right)}^{\mathrm{nT}}\right)}^{\frac{1}{\mathrm{nT}}}}$$

where q_mT_ is the maximum adsorption capacity [mmol/g], b_T_ is the Toth constant [bar^−1^], n_T_ is the heterogeneity factor.

### 1.4. Sips Isotherm

The Sips model is commonly used for the specification of the heterogeneous adsorbents like activated carbons [27,28]. It is reduced to the Freundlich model at low adsorbate concentrations, and at high adsorbate concentrations, it is similar to the Langmuir model [29] and can be expressed by an Equation (4):

(4) $$\;q=\frac{q_{\mathrm{mS}}b_{S}p^{n_{S}}}{1+b_{S}p^{n_{S}}},$$

where q_mS_ is the maximum adsorption capacity [mmol/g], *b_S_* is the Sips constant [bar^−1^], *n_S_* is the heterogeneity factor.

### 1.5. Fritz-Schlunder Isotherm

Fritz and Schlunder elaborated an empirical Equation (5) that is suitable for an extended range of experimental results because of many coefficients in the isotherm [30]:

(5) $$\;q=\frac{q_{\mathrm{mFS}\;}b_{\mathrm{FS}}p}{1+q_{\mathrm{mFS}}{\mathrm{pn}}_{\mathrm{FS}}}\;$$

where q_mFS_ is the maximum adsorption capacity [mmol/g], b_FS_ is the Fritz-Schlunder constant [bar^−1^], n_FS_ is the Fritz-Schlunder model exponent.

### 1.6. Radke-Prausnitz Isotherm

The Radke-Prausnitz model has several significant properties that make it the preferred choice for most adsorption systems with low adsorbate concentrations. At a low adsorbate concentration, the isotherm model reduces to a linear isotherm. At a high adsorbate concentration, it approaches the Freundlich isotherm, and when n_RP_ = 0, it becomes a Langmuir isotherm. Another essential property of this isotherm is that it gives a good fit over an extended range of adsorbate concentrations. Radke-Prausnitz equation can be expressed as [31]:

(6) $$\;q=\frac{q_{\mathrm{mRP}}b_{\mathrm{RP}}p}{{\left(1+b_{\mathrm{RP}}p\right)}^{n_{\mathrm{RP}}}},$$

where q_mRP_ is the maximum adsorption capacity [mmol/g], b_RP_ is the Radke-Prausnitz constant [bar^−1^], n_RP_ is Radke-Prausnitz model exponent.

### 1.7. Unilan Isotherm

Unilan model (7) assumes a heterogeneous surface and almost continuous energy distribution of site yields [32]:

(7) $$q=\frac{q_{\mathrm{mU}}}{2s}\ln\left(\frac{1+b_{U}\exp^{\left(s\right)}\cdot p}{1+b_{U}\exp^{\left(-s\right)}\cdot p}\right)$$

where q_mU_ is the maximum adsorption capacity [mmol/g], b_U_ is the Unilan constant [bar^−1^], s is the constant dependent on the difference between the minimum and maximum adsorption energy.

### 1.8. Temkin Isotherm

This is an empirical two-parameter model for the isotherm of adsorption on a heterogeneous solid. The isotherm corresponds to the continuous, unlimited energy distribution of adsorption sites. The isotherm equation assumes that the heat of adsorption of all molecules in the layer decreases linearly from the adsorbent-adsorbate interaction, and the adsorption is characterized by an equable distribution of the bonding energy. The Temkin equation can be described as (8) [33]:

(8) $$q=\frac{\mathrm{RT}}{b_{Te}}\ln A_{Te}p$$

where R is the universal gas constant [J/(mol·K)], *b_Te_* is the Temkin-Pyzhev constant connected with the heat of sorption [J/mol], *A_Te_* is the Temkin-Pyzhev constant.

### 1.9. Dubinin-Radushkevich Isotherm

The Dubinin-Radushkevich model is connected with adsorption energy. It mainly concerns adsorption in micropores. It was assumed that the pore size distribution is heterogeneous and can be described by a Gaussian function. Volumetric filling of micropores was assumed as a result of the increased adsorption potential resulting from the overlapping of the adsorption potentials close to the walls. In the case of a flat surface, increased heat of adsorption occurs at the creation of a monolayer, and in the case of micropores, the adsorption density is increased when filling the micropores. The Dubinin-Radushkevich equation can be described as follows (9) [34]:

(9) $$q=q_{\mathrm{mDR}}e^{-A{\left(\ln1+\frac{1}{p}\right)}^{2}}$$

where A is defined by Equation (10):

(10) $$A=b_{DR}R^{2}T^{2}$$

where *b_DR_* is the Dubinin-Radushkevich constant connected with the heat of sorption [mol^2^/kJ^2^], q_mDR_ is the Dubinin-Radushkevich constant connected with amount of adsorbed gas.

### 1.10. Jovanovic Isotherm

The Jovanovic model retains the assumptions of the Langmuir model taking into account the possibility of additional interactions resulting in the multi-layer sorption effect. The Jovanovich equation can serve as the local isotherm in the general integral equation describing adsorption on a heterogeneous surface (11) [35]:

(11) $$q=q_{\mathrm{mJ}}\left(1-e^{-b_{J}p}\right)$$

where *^bJ^* is the Jovanovich constant.

The nonlinear optimization was used to determine the fitted isotherm. Nonlinear model can be an influential substitute to linear regression because it involves the most flexible curve-fitting functionality. Using nonlinear regression, the sum of the squares of the errors (SSE) must be minimized by an iterative method over the entire range of data. The other error functions such as the hybrid fractional error function (HYBRID), the average relative error (ARE), the Marquardt’s percent standard deviation (MPSD), and the sum of the absolute errors (SAE) can be also utilized to expect the best isotherm. The smaller error of the estimate indicates the more accurate prediction. The best set of parameters for each isotherm was selected using the sum of the normalized error (SNE). Detailed information about error functions was defined by Equations (12)–(16):

The Sum of the Squares of the Errors (SSE) [36]:

(12) $$\mathrm{SSE}=\overset{n}{\underset{i=1}{\sum }}{\left(q_{e,calc}-q_{e,\exp}\right)}_{i}^{2}$$

where *q_e,calc_* is the calculated adsorption capacity [mmol/g], *q*_*e*,exp_ is the experimentally measured adsorption capacity [mmol/g].

The Hybrid Fractional Error Function (HYBRID) [37]:

(13) $$\mathrm{HYBRID}=\frac{100}{1-p}\overset{n}{\underset{i=1}{\sum }}{\left[\frac{{\left(q_{e,calc}-q_{e,\exp}\right)}^{2}}{q_{e,\exp}}\right]}_{i}$$

The Average Relative Error Function (ARE) [38]:

(14) $$\mathrm{ARE}=\frac{100}{1-p}\overset{n}{\underset{i=1}{\sum }}{\left[\frac{q_{e,calc}-q_{e,\exp}}{q_{e,\exp}}\right]}_{i}$$

The Marquardt’s Percent Standard Deviation (MPSD) [39]:

(15) $$\mathrm{MPSD}=100\sqrt{\frac{1}{n-p}\overset{n}{\underset{i=1}{\sum }}{\left(\frac{q_{e,calc}-q_{e,\exp}}{q_{e,\exp}}\right)}_{i}{}^{2}}$$

The Sum of the Absolute Errors Function (SAE) [40]:

(16) $$\mathrm{SAE}=\overset{n}{\underset{i=1}{\sum }}{\left(q_{e,calc}-q_{e,\exp}\right)}_{i}^{}$$

The aim of each error function is to obtain various set of isotherm parameters, thus, the optimal parameters are hard for straight interpretation. It may also happen that on the basis of different error functions, another model should be recognized as the best. Thus, the selection of error function could influence on the obtained isotherm parameters. The sum of the normalized errors (SNE) can be applied to the important parameters comparison [36]. Shortly, to calculate SNE, the values of the errors obtained for each error function for every group of isotherm constants were divided by the maximum errors for that error function. A function was chosen on the basis of the lowest number of SNE with the best defined empirical results.

The goal of the present study is to examine the CO_2_ adsorption over the activated carbons derived from beet molasses connected with the research of the influence of the isotherm kind and the model used to calculate its parameters on the calculations by the two and three parametric models taking into discuss the error functions.

The novelty of the work was the application of solid KOH as an activator. All the carbon sources described in the literature are solid-state. While, molasses is liquid and was described only by Legrouri et al. [41] and our group [42]. Legrouri et al. [41] used sulphuric acid as an activator. In our previous research [42] we dried and ground molasses in order to get powder moreover KOH solution was applied. The new method presented here is much simpler and inexpensive (no drying necessary).

## 2. Materials and Methods

Chemical activation of beet molasses was carried out with the use of solid potassium hydroxide. Liquid molasses was weighed into a plastic cup, and then potassium hydroxide was added in such an amount that the mass ratio of molasses to activator was 1:1. Then, the material was vigorously mixed until the raw material was clearly saturated with solid potassium hydroxide and left at ambient temperature for 3 h. After this time, the impregnated material was placed in a laboratory dryer (20 h, 200 °C). The carbonaceous precursor impregnated in this way was carbonized. A physical activation process was conducted in a tubular reactor kept for 1 h in electrical furnace in the temperature range of 650–800 °C and the temperature was increased 10 °C per minute to a chosen value. The process was carried out in the nitrogen-carbon dioxide atmosphere (flow rate equal to 18 dm^3^/h, flow of the carbon dioxide 5 dm^3^/h). The activation process parameters like, time, N_2_-CO_2_ flow rate, and the heating rate of furnace in all the experiments were identical. They were assumed, based on many previous tests, to result in the best settings ensuring the maximum enhancement of the surface area of studied carbons. The derived activated carbon containing the decomposition products of potassium hydroxide or potassium carbonate were rinsed with deionized water to attain a neutral reaction. When the sample was evaporated, the activated carbon was flooded with 1 mol/dm^3^ HCl solution and was left behind for 20 h. In the following stage, carbons were rinsed with deionized water until complete removal of chloride ions. Then samples were dried at temperature of 110 °C for 16 h. The activated carbons were denoted as: M1_KOH_650_18N2_5CO2, M1_KOH_700_18N2_5CO2, M1_KOH_750_18N2_5CO2, M1_KOH_800_18N2_5CO2, where: M1 is beet molasses, KOH is an activating agent, 650, 700, 750, 800 is an activation temperature, and 18N2_5CO2 is the gaseous activating atmosphere. All activated carbons were characterized by nitrogen adsorption at −196 °C by means of Sorption Surface Area and Pore Size Analyzer (ASAP 2460, Micrometrics, Novcross, USA). To remove the contaminants from samples, the adsorption measurements were preceded by heating at temperature of 250 °C for 12 h with the heating rate of 1°/min under the reduced pressure thanks to the constant operation of pump. From N_2_ sorption isotherms, the following parameters describing the porous structure have been obtained:

- Surface area (S_BET_) estimated on the basis of the BET equation with the partial pressure in the range of/*p*_0_ = 0.05–0.2. This range was pointed independently for each material so that a linearity of function (17) were fulfilled:

(17) $$f\left(\frac{p}{p_{0}}\right)=\frac{1}{W\left(\frac{p}{p_{0}}-1\right)}$$

where *W* is the mass of gas adsorbed at a relative pressure *p/p*_0_, *p* is the nitrogen pressure, *p*_0_ is equal to 1.01 bar;

- Total pore volume (V*_p_*_,N2_) calculated from the maximum adsorption of nitrogen vapor for *p*/*p*_0_ = 0.99;
- Pores in a range of micropores (V_mic,N2_) and mesopores were evaluated using N_2_ analysis at −196 °C temperature by the DFT method (density functional theory).

The N_2_ adsorption isotherm at −196 °C gives data about the micropore structure with a size over 1.5 nm and the mesopores, and partly macropores. The CO_2_ adsorption measurements were studied at temperature of 0 °C, under pressure to 1 bar using ASAP. So as to control the experiment temperature, investigated were located in a thermostat. Before the CO_2_ adsorption measurements, the activated carbons were outgassed at temperature of 250 °C for 12 h.

## 3. Results and Discussion

**Figure 1:**
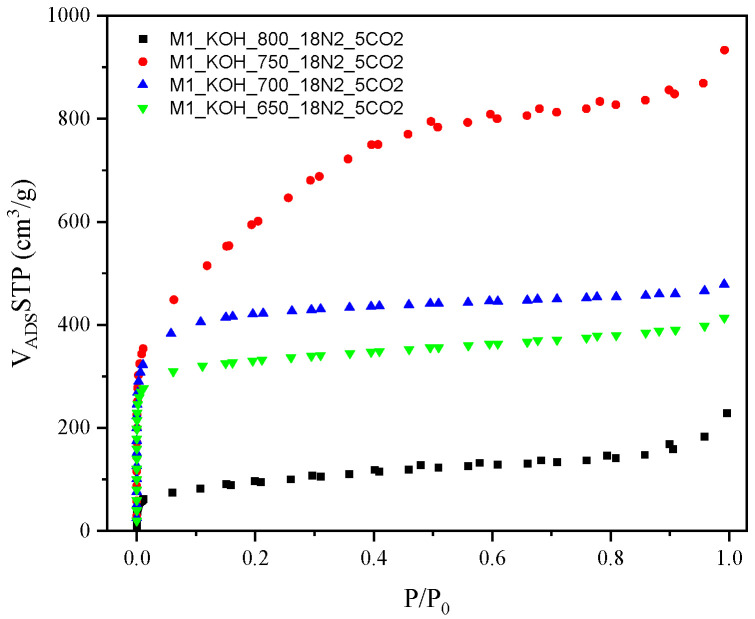
The adsorption-desorption isotherms of N_2_ for activated carbons.

The results of undermentioned adsorption-desorption isotherms of N_2_ on the examined activated carbons are shown in Figure 1.

The isotherms established a high adsorption of N_2_ at low relative pressure that is representative for the microporous samples. A high N_2_ adsorption at a low relative pressure (under 0.1 *p*/*p*_0_) designates high volume of the micropores with a thin pore size distribution. It was observed, that the nitrogen adsorption measured at temperature −196 °C meaningfully increased in case of all carbon samples along with increase of an activation temperature during the thermal treatment, however, with one exception i.e., the lowest nitrogen capacity was achieved for carbon activating at the highest temperature (800 °C).

By International Union of Pure and Applied Chemistry (IUPAC) classification, the nitrogen adsorption isotherms correspond to the Type I at the first range (low value of the relative pressure *p*/*p*_0_), while in the medium and higher range to the Type IV. A representative feature of the Type IV isotherm is the existence of sharply formed hysteresis loop which is related with capillary condensation occurring in the area of mesopores. The isotherms established the hysteresis loop of the Type IV. It was concluded that the capillary condensation in the mesopores occurs in the range of relative pressure *p*/*p*_0_ = 0.45–1, for all four samples, designating the presence of mesopores.

**Figure 2:**
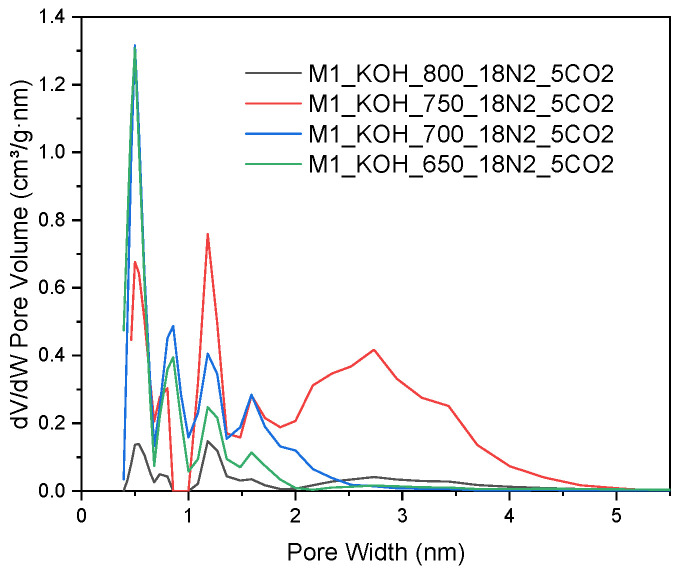
The pores size distribution of the activated carbons, N_2_ adsorption at −196 °C.

Taking into account an analysis of pore size distribution, more comprehensive information can be found regarding the structure of the adsorption over tested materials. In order to investigate the relationships between the pore size of the studied carbons and a temperature of activation process, an analysis of the size distribution of the activated carbons based on the N_2_ adsorption was performed. The pore distribution shown in Figure 2 directs for the fact that all samples in addition to a relatively well developed microporosity indicates the advanced mesoporosity as well. The used method provides information on the porosity for pores in the range from 0.35 to 300 nm, depending on the used adsorbate. However, in Figure 2 only pores up to 5 nm are presented, as there were no larger pores in the tested activated carbons.

The textural properties of all samples were compiled in Table 1. In case of samples M1_650_18N2_5CO2, M1_700_18N2_5CO2, M1_750_18N2_5CO2, higher surface areas and pore volumes were obtained with increasing activation temperature. However, for sample M1_800_18N2_5CO2 the tendency is the differing, as observed in Table 1. The largest BET surface area (2075 m^2^/g) attained the M1_750_18N2_5CO2 carbon. In the other hand, the most microporous material with the micropore volume of 0.53 cm^3^/g were M1_700_18N2_5CO2 carbon.

**Figure 3:**
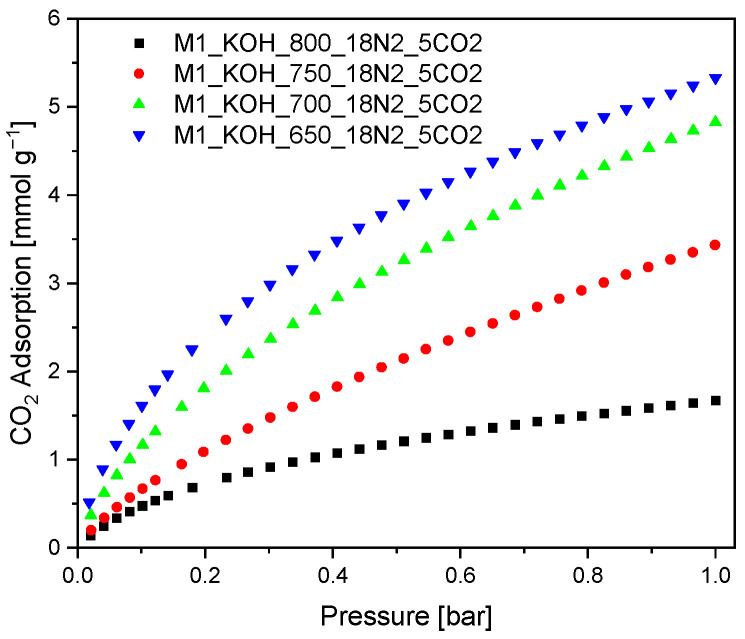
CO_2_ adsorption isotherms measured at 0 °C.

The CO_2_ adsorption on the surface of the activated carbons was measured at temperature of 0 °C under pressure of 1 bar. The experimental CO_2_ capacity at 0 °C are given in Figure 3.

It was evidenced, that the CO_2_ adsorption capacity at temperature 0 °C increased along with decreasing carbonization temperature. These results are surprising because they are contrary to the literature reports [43], where the CO_2_ adsorption efficiency increases along with increasing: specific surface area, total pore volume as well as micropore volume. Therefore, it can be concluded that in the case of studied activated carbons the key role is played by pores with diameter in the range from 0.3 to 0.6 nm with ignoble participation of the larger pores. 

Table 2 summarizes the results of adsorption CO_2_ on activated carbons produced from various carbon precursors.

All isotherms match to type I of IUPAC classification, characteristic for microporous adsorbents. Experimental CO_2_ adsorption isotherms constituted the basis for calculating equation parameters in all models.

The sets of CO_2_ adsorption isotherm parameters and error functions with SNE are compiled in Table 3, Table 4, Table 5, Table 6, Table 7, Table 8, Table 9, Table 10, Table 11 and Table 12. The comparison of the SNE was undertaken and, hence, the isotherm constants which present the closest fitting to the measured data were attained.

The bold marked numbers in Table 3, Table 4, Table 5, Table 6, Table 7, Table 8, Table 9, Table 10, Table 11 and Table 12 symbolize the minimum SNE for each isotherm and each activated carbon, while the underlined numbers designate the lowest SNE value from all the isotherms and the optimum parameters set for each activated carbon.

The parameters fitting results to Langmuir model are exposed in Table 3.

Presented constants were estimated by nonlinear regression making use of the different error functions. The values of constants q_mL_ and b_L_ are quite similar. Langmuir isotherm does not provide a good model for the CO_2_ adsorption over all activated carbons. As indicated by the SNE, the parameter set that produces the best overall Langmuir fit are HYBRID for all four activated carbons.

The Freundlich isotherms constants and error functions are shown in Table 4.

Based on SNE, the ARE for M1_800_18N2_5CO2, and HYBRID for M1_650_18N2_5CO2, M1_700_18N2_5CO2, M1_750_18N2_5CO2 give the best Freundlich fit. Nevertheless, the best Freundlich fit cannot be acceptable.

The fitting parameters to Sips model are shown in Table 5.

The SNE indicated that the HYBRID gives the best Sips fit.

The Toth isotherms constants and error functions are shown in Table 6.

The SNE specified that the MPSD for M1_650_18N2_5CO2, SAE for M1_750_18N2_5CO2, and HYBRID for the rest two activated carbons give the best Toth fit.

The fitting parameters to Unilan model are shown in Table 7.

The SNE specified that the ARE for M1_650_18N2_5CO2, and HYBRID for the rest of the activated carbons give the best Unilan fit.

The Fritz-Schlunder isotherms constants and error functions are shown in Table 8.

The SNE specified that the SAE for M1_750_18N2_5CO2, and HYBRID for the rest of the activated carbons give the best Fritz-Schlunder fit.

The Temkin isotherms constants and error functions are shown in Table 9.

The SNE specified that the HYBRID for all activated carbons gives the best Temkin fit.

The Dubinin-Raduskevich isotherms constants and error functions are shown in Table 10.

The SNE specified that the HYBRID for the all activated carbons gives the Dubinin-Raduskevich fit.

The Jovanovich isotherms constants and error functions are shown in Table 11.

The SNE specified that the ARE for M1_700_18N2_5CO2, and HYBRID for the rest of the two activated carbons give the best Jovanovivh fit.

The Radke-Prausnitz isotherms constants and error functions are shown in Table 12.

**Figure 4:**
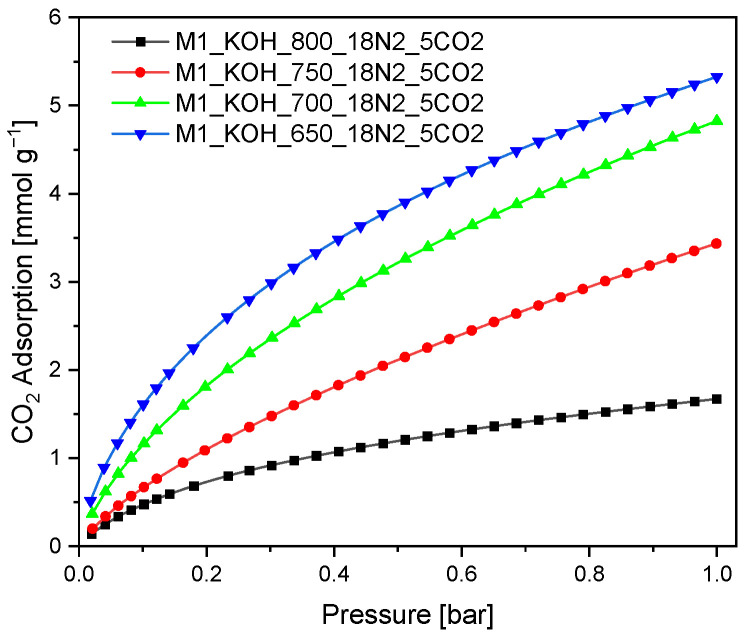
CO_2_ adsorption isotherms measured at 0 °C. The symbols express empirical results, lines were obtained based on the Radke-Prausnitz model (a function was chosen on the basis of the lowest value of SNE which the best characterizes experimental values).

The SNE specified that the MPSD for M1_750_18N2_5CO2, the ARE for M1_800_18N2_5CO2 and HYBRID for the two activated carbons give the best Radke-Prausnitz fit. The three constants q_mRP_, b_RP_, n_RP_ are comparable over the whole range of error functions. The SNE for M1_650_18N2_5CO2, M1_800_18N2_5CO2 were the lowest of all the studied models. The Radke-Prausnitz equation gives a rational approximation to the optimum parameter set. The theoretical Radke-Prausnitz isotherms and experimental data are presented in Figure 4.

The Radke-Prausnitz model is advised for the analysis of the empirical data. A similar result can be concluded based on Figure 4. The experimental adsorption isotherm matches quite well with Radke-Prausnitz equation model regardless of the error function.

## 4. Conclusions

The results of the CO_2_ adsorption at 0 °C on four activated carbons derived from the raw beet molasses and activated with solid KOH show that these carbonaceous materials can be interesting for CO_2_ capture enhancement. The obtained specific surface area is as high as 2075 m^2^g^−1^, and total pore volume up to 1.44 cm^3^g^−1^ corresponding to the activated carbon labeled as M1_750_18N2_5CO2. Moreover, it was evidenced, that the CO_2_ adsorption capacity at temperature 0 °C increased along with decreasing carbonization temperature. The activated carbons marked as M1_650_18N2_5CO2 can adsorb as much CO_2_ as 5.4 mmolg^−1^ at 0 °C and 1 bar.

The examined equilibrium adsorption results were calculated and evaluated according to ten different isotherms and five different optimization and error functions. Based on the sum of normalized errors the comparison of error function was made, and the best isotherm equation was found. The Radke-Prausnitz gives the best estimation as it is the most appropriate model with the empirical data.

## Figures and Tables

**Table 1:** Textural parameters for activated carbons, derived from N_2_ adsorption isotherms at −196 °C.

Sample	S_BET_ [m^2^/g]	V_p,N2_ [cm^3^/g]	V_mic,N2_ [cm^3^/g]
M1_650_18N2_5CO2	1247	0.64	0.4
M1_700_18N2_5CO2	1575	0.71	0.53
M1_750_18N2_5CO2	2075	1.44	0.46
M1_850_18N2_5CO2	326	0.35	0.07

**Table 2:** Langmuir CO_2_ adsorption of various activated carbons at 1 bar and 0 °C.

Material	CO_2_ Adsorption at 0 °C [mmol/g]	Refs.
organic framework polymers	2.9	[44]
activated carbon xerogels	4.9	[45]
Mg and N-doped mesoporous carbon	3.7	[46]
waste wool-derived N-doped hierarchical porous carbon	3.7	[47]
activated carbon monoliths	9.1	[48]
polyaniline-graphene oxides	3.2	[49]
phenolic resin-derived carbon spheres	8.9	[50]
KOH activated carbon derived from raw molasses	5.4	this work

**Table 3:** Langmuir isotherms constants with error analysis *.

	SSE	HYBRID	ARE	MPSD	SAE
M1_650_18N2_5CO2					
q_mL_	7.2707	6.7818	6.6419	6.1657	7.5580
b_L_	2.4183	2.8826	2.9746	3.6483	2.1633
SSE	0.5058	0.6586	0.7666	1.4430	0.5884
HYBRID	1.1769	0.9676	0.9984	1.3673	1.5786
ARE	5.9061	5.4549	5.4176	5.9606	6.4050
MPSD	11.1292	9.0957	8.9495	7.9138	12.8738
SAE	3.39919	3.9596	4.1155	5.5469	3.1862
SNE	3.4954	**3.3413**	3.4467	4.4115	3.9822
M1_700_18N2_5CO2					
q_mL_	8.0398	7.2929	7.0237	6.3559	8.2289
b_L_	1.3988	1.6943	1.7979	2.2240	1.3194
SSE	0.2922	0.4024	0.5183	1.0479	0.3200
HYBRID	0.8823	0.7123	0.7452	1.0937	1.0465
ARE	5.8638	5.4440	5.2687	5.8189	6.1797
MPSD	11.0699	8.9443	8.5690	7.5538	12.0371
SAE	2.5697	3.1291	3.3528	4.6078	2.4829
SNE	3.5118	**3.3383**	3.4842	4.5691	3.8010
M1_750_18N2_5CO2					
q_mL_	7.2701	6.5517	6.2432	5.3984	7.7763
b_L_	2.4183	1.0075	1.0685	1.3850	0.7605
SSE	0.5058	0.1395	0.1987	0.4368	0.1048
HYBRID	1.1769	0.3865	0.4222	0.6498	0.5752
ARE	5.9061	5.2092	5.1408	5.5799	6.0437
MPSD	11.1292	8.7370	8.5614	7.2350	11.6905
SAE	3.3992	1.8368	1.9745	2.9106	1.4299
SNE	4.9292	**2.7539**	2.9155	3.8141	3.1167
M1_800_18N2_5CO2					
q_mL_	2.3634	2.2177	2.1264	2.0454	2.4152
b_L_	2.1749	2.5319	2.7403	3.0550	2.0372
SSE	0.0354	0.0463	0.0669	0.0955	0.0392
HYBRID	0.2405	0.1916	0.2128	0.2700	0.3046
ARE	5.0117	4.5123	4.3913	4.6334	5.3820
MPSD	8.8630	6.8759	6.3129	5.7975	10.0703
SAE	0.9143	1.0524	1.1685	1.3988	0.8799
SNE	3.6249	**3.3866**	3.6769	4.3230	4.0390

* Standard uncertainties of all constants are equal to 0.001, uncertainties of all errors equal to 0.0001 (0.95 level of confidence).

**Table 4:** Freundlich isotherms constants with error analysis *.

	SSE	HYBRID	ARE	MPSD	SAE
M1_650_18N2_5CO2					
q	5.4205	5.4856	5.5150	5.5975	5.3964
b_F_	0.5139	0.5354	0.5367	0.5603	0.5012
SSE	0.1401	0.1980	0.2211	0.4431	0.1625
HYBRID	0.3874	0.2846	0.2923	0.4152	0.5647
ARE	3.2034	2.8662	2.8350	3.2776	3.6642
MPSD	7.2039	5.1631	5.1589	4.2316	8.8241
SAE	1.7629	2.1561	2.1921	3.0892	1.6738
SNE	3.2636	**3.0160**	3.0845	4.1093	3.9085
M1_700_18N2_5CO2					
q	4.8747	4.9154	4.9327	4.9916	4.8676
b_F_	0.6134	0.6293	0.6314	0.6487	0.6054
SSE	0.0351	0.0542	0.0638	0.1471	0.0414
HYBRID	0.1387	0.0986	0.1008	0.1565	0.1980
ARE	2.1842	1.9252	1.9129	2.2214	2.4765
MPSD	5.1089	3.6259	3.5209	2.8951	6.1199
SAE	0.8572	1.1171	1.1772	1.7416	0.7960
SNE	3.1485	**2.8783**	2.9663	4.1604	3.7383
M1_750_18N2_5CO2					
q	3.4494	3.4629	3.4653	3.4932	3.4476
b_F_	0.7105	0.7184	0.7183	0.7294	0.7080
SSE	0.0025	0.0044	0.0045	0.0161	0.0027
HYBRID	0.0198	0.0141	0.0142	0.0254	0.0243
ARE	1.0365	0.9412	0.9334	1.1573	1.1344
MPSD	2.6721	1.9611	1.9862	1.5462	2.9536
SAE	0.2122	0.3164	0.3168	0.5735	0.2036
SNE	3.1073	**2.8550**	2.8730	4.5235	3.4631
M1_800_18N2_5CO2					
q	1.7039	1.7311	1.7350	1.7808	1.6913
b_F_	0.5361	0.5655	0.5627	0.6016	0.5148
SSE	0.0199	0.0299	0.0293	0.0754	0.0257
HYBRID	0.2135	0.1537	0.1561	0.2358	0.3477
ARE	4.2889	3.8856	3.8502	4.5703	5.1219
MPSD	10.3752	7.3861	7.7001	5.9423	13.3000
SAE	0.6473	0.8396	0.8150	1.2886	0.5973
SNE	2.9977	2.8043	**2.8004**	4.0172	3.8045

* Standard uncertainties of all constants are equal to 0.001, uncertainties of all errors equal to 0.0001 (0.95 level of confidence).

**Table 5:** Sips isotherms constants with error analysis *.

	SSE	HYBRID	ARE	MPSD	SAE
M1_650_18N2_5CO2					
q_mS_	14.5112	13.5991	13.6306	12.6177	13.6362
b_S_	0.5774	0.6389	0.6348	0.7201	0.6347
n_S_	0.6698	0.6835	0.6811	0.6985	0.6823
SSE	0.0021	0.0032	0.0040	0.0084	0.0039
HYBRID	0.0079	0.0055	0.0063	0.0084	0.0062
ARE	0.3765	0.3752	0.3598	0.4799	0.3625
MPSD	1.1333	0.7660	0.8467	0.6191	0.7971
SAE	0.1846	0.2621	0.2434	0.4270	0.2415
SNE	3.4052	**3.1082**	3.2932	4.5463	3.2227
M1_700_18N2_5CO2					
q_mS_	22.6789	19.8826	19.9379	17.2029	17.4800
b_S_	0.2698	0.3190	0.3172	0.3856	0.3777
n_S_	0.7046	0.7193	0.7165	0.7353	0.7366
SSE	0.0016	0.0025	0.0029	0.0073	0.0061
HYBRID	0.0076	0.0051	0.0059	0.0083	0.0091
ARE	0.4527	0.4383	0.4209	0.5216	0.5614
MPSD	1.2981	0.8558	0.9880	0.6795	0.7877
SAE	0.1736	0.2488	0.2427	0.3972	0.3692
SNE	3.2912	**2.9670**	3.1687	4.3638	4.3763
M1_750_18N2_5CO2					
q_mS_	42.7761	32.7762	32.7525	24.6378	24.7621
b_S_	0.0873	0.1168	0.1168	0.1609	0.1602
n_S_	0.7457	0.7567	0.7550	0.7697	0.7728
SSE	0.0004	0.0006	0.0008	0.0022	0.0018
HYBRID	0.0031	0.0021	0.0023	0.0036	0.0043
ARE	0.3799	0.3564	0.3413	0.4385	0.4809
MPSD	1.1134	0.7421	0.8305	0.5790	0.7074
SAE	0.0834	0.1195	0.1201	0.2177	0.2004
SNE	3.0586	**2.7163**	2.9021	4.2867	4.3704
M1_800_18N2_5CO2					
q_mS_	4.0852	3.6207	3.6222	3.2118	3.9718
b_S_	0.6867	0.8426	0.8377	1.0460	0.7162
n_S_	0.7222	0.7558	0.7507	0.7898	0.7249
SSE	0.0011	0.0017	0.0019	0.0044	0.0013
HYBRID	0.0130	0.0086	0.0095	0.0131	0.0135
ARE	0.9887	0.9161	0.8855	1.0281	0.9617
MPSD	2.6725	1.6678	1.8912	1.2971	2.7228
SAE	0.1519	0.2034	0.1936	0.2998	0.1507
SNE	3.6710	**3.2133**	3.3430	4.4471	3.7447

* Standard uncertainties of all constants are equal to 0.001, uncertainties of all errors equal to 0.0001 (0.95 level of confidence).

**Table 6:** Toth isotherms constants with error analysis *.

	SSE	HYBRID	ARE	MPSD	SAE
M1_650_18N2_5CO2					
q_mT_	40.2218	40.3647	40.3647	40.1583	40.1583
b_T_	2.3392	2.3385	2.3385	2.3413	2.3413
n_T_	0.2862	0.2858	0.2858	0.2864	0.2864
SSE	0.0001	0.0001	0.0001	0.0001	0.0001
HYBRID	0.0001	0.0001	0.0001	0.0001	0.0001
ARE	0.0565	0.0569	0.0568	0.0564	0.0563
MPSD	0.0761	0.0764	0.0764	0.0759	0.0760
SAE	0.0538	0.0540	0.0539	0.0536	0.0534
SNE	4.9728	4.9859	4.9858	**4.9721**	4.9749
M1_700_18N2_5CO2					
q_mT_	280.7508	213.6424	213.4485	179.3629	179.3701
b_T_	0.2713	0.3115	0.3126	0.3432	0.3405
n_T_	0.2058	0.2186	0.2185	0.2272	0.2276
SSE	0.0002	0.0003	0.0004	0.0007	0.0006
HYBRID	0.0007	0.0005	0.0005	0.0008	0.0008
ARE	0.1406	0.1367	0.1340	0.1581	0.1646
MPSD	0.3931	0.2351	0.2410	0.2058	0.2178
SAE	0.0611	0.0884	0.0887	0.1235	0.1186
SNE	3.5700	**3.3008**	3.3423	4.4839	4.3731
M1_750_18N2_5CO2					
q_mT_	44.1893	11.0799	26.7999	7608.1991	11.7956
b_T_	0.2372	0.7164	0.4260	0.0061	0.5806
n_T_	0.3975	0.6731	0.4255	0.1478	0.7275
SSE	0.0151	0.0796	0.0824	0.0010	0.0573
HYBRID	0.0775	0.1974	0.1160	0.0014	0.3323
ARE	2.2396	3.6127	2.1730	0.2527	4.5300
MPSD	4.5081	6.0579	2.9631	0.2947	9.1518
SAE	0.5953	1.3620	1.1877	0.1408	1.0811
SNE	0.8445	5.0000	4.0287	0.9757	**0.7624**
M1_800_18N2_5CO2					
q_mT_	40.2680	6.9233	6.9180	5.8147	6.2670
b_T_	2.2444	1.9293	1.9604	2.0420	1.9871
n_T_	0.1937	0.3999	0.3980	0.4373	0.4206
SSE	0.0117	0.0005	0.0006	0.0012	0.0008
HYBRID	0.1369	0.0024	0.0026	0.0035	0.0028
ARE	3.3533	0.4828	0.4670	0.5078	0.4806
MPSD	8.0099	0.8278	0.9158	0.6402	0.6801
SAE	0.4599	0.1116	0.1077	0.1527	0.1297
SNE	5.0000	**0.5523**	0.5612	0.6904	0.6021

* Standard uncertainties of all constants are equal to 0.001, uncertainties of all errors equal to 0.0001 (0.95 level of confidence).

**Table 7:** Unilan isotherms constants with error analysis *.

	SSE	HYBRID	ARE	MPSD	SAE
M1_650_18N2_5CO2					
q_mU_	29.7886	29.5362	29.5515	26.6503	29.1481
b_U_	0.0103	0.0078	0.0081	0.0102	0.0143
s	6.9109	7.3464	7.2779	7.2802	6.4724
SSE	0.1470	0.1987	0.1875	0.4639	0.1772
HYBRID	0.4034	0.3197	0.3256	0.4663	0.5772
ARE	3.2658	3.0143	2.9978	3.4529	3.6737
MPSD	7.0229	5.5311	5.8026	4.7159	8.3886
SAE	1.7841	2.1529	2.0561	3.1704	1.6818
SNE	3.3046	3.1412	**3.1246**	4.3099	3.9126
M1_700_18N2_5CO2					
q_mU_	30.9186	30.0303	29.8495	29.1766	34.5919
b_U_	0.0160	0.0122	0.0098	0.0075	0.0100
s	5.6808	6.1527	6.4603	6.9048	6.0610
SSE	0.1466	0.2084	0.3054	0.5367	0.1629
HYBRID	0.4840	0.3736	0.4056	0.5762	0.6234
ARE	4.2275	3.8511	3.7710	4.1278	4.6160
MPSD	8.5416	6.5779	6.0066	5.4379	9.6978
SAE	1.8157	2.2449	2.5351	3.2897	1.7379
SNE	3.3981	**3.1826**	3.4266	4.3793	3.8317
M1_750_18N2_5CO2					
q_mU_	33.7655	31.5422	31.3298	28.0135	37.9369
b_U_	0.0059	0.0045	0.0040	0.0033	0.0024
s	5.9507	6.4701	6.6122	7.1095	6.8951
SSE	0.0662	0.0989	0.1150	0.3039	0.0707
HYBRID	0.3558	0.2738	0.2776	0.4612	0.3428
ARE	4.7832	4.3062	4.2458	4.5472	4.6589
MPSD	9.4832	7.3899	7.1442	5.9847	9.2592
SAE	1.2297	1.5447	1.6097	2.4078	1.2398
SNE	3.5001	**3.2402**	3.2897	4.5817	3.4413
M1_800_18N2_5CO2					
q_mU_	11.0358	10.8140	10.7299	10.5332	11.6580
b_U_	0.0042	0.0037	0.0037	0.0031	0.0031
s	7.6246	7.8839	7.9095	8.1774	7.8759
SSE	0.0086	0.0114	0.0129	0.0229	0.0095
HYBRID	0.0622	0.0472	0.0481	0.0664	0.0822
ARE	2.4952	2.1466	2.1102	2.2393	2.7443
MPSD	4.7396	3.4608	3.3382	2.8569	5.5399
SAE	0.4521	0.5132	0.5287	0.6803	0.4376
SNE	3.5624	**3.2344**	3.2987	4.1402	4.0579

* Standard uncertainties of all constants are equal to 0.001, uncertainties of all errors equal to 0.0001 (0.95 level of confidence).

**Table 8:** Fritz-Schlunder isotherms constants with error analysis *.

	SSE	HYBRID	ARE	MPSD	SAE
M1_650_18N2_5CO2					
q_mFS_	7.1453	7.7204	7.7204	8.2684	6.6997
b_FS_	6.0801	6.0346	6.0345	6.0045	6.1248
n_FS_	0.6237	0.6114	0.6107	0.5990	0.6327
SSE	0.0018	0.0027	0.0029	0.0059	0.0024
HYBRID	0.0057	0.0038	0.0039	0.0055	0.0111
ARE	0.3647	0.3350	0.3324	0.3670	0.4441
MPSD	0.9164	0.5601	0.5688	0.4408	1.3448
SAE	0.1993	0.2577	0.2611	0.3621	0.1815
SNE	2.8809	**2.6845**	2.7340	3.6452	3.9137
M1_700_18N2_5CO2					
q_mFS_	5.5288	5.5968	5.6070	5.6899	5.4502
b_FS_	5.7066	5.6944	5.6921	5.6828	5.7161
n_FS_	0.5021	0.5008	0.5008	0.4981	0.5040
SSE	0.0002	0.0001	0.0001	0.0002	0.0001
HYBRID	0.0002	0.0002	0.0002	0.0002	0.0003
ARE	0.0796	0.0656	0.0637	0.0651	0.0885
MPSD	0.1439	0.1044	0.1012	0.0871	0.2036
SAE	0.0517	0.0470	0.0466	0.0579	0.0455
SNE	4.1217	**3.3694**	3.3437	3.9239	4.4062
M1_750_18N2_5CO2					
q_mFS_	5.8347	14.4531	6.0042	5.0523	5.3332
b_FS_	4.0263	3.6913	4.0110	4.1119	4.0805
n_FS_	0.3585	0.3113	0.3560	0.3718	0.3650
SSE	0.0001	0.0016	0.0001	0.0002	0.0001
HYBRID	0.0003	0.0060	0.0004	0.0004	0.0003
ARE	0.1371	0.5998	0.1433	0.1379	0.1234
MPSD	0.3185	1.3301	0.3566	0.1701	0.2055
SAE	0.0444	0.1904	0.0441	0.0708	0.0475
SNE	0.8154	5.0000	0.8673	0.9292	**0.7340**
M1_800_18N2_5CO2					
q_mFS_	5.0158	4.8622	4.8567	4.7483	5.0885
b_FS_	2.0027	2.0115	2.0092	2.0170	1.9981
n_FS_	0.6512	0.6586	0.6605	0.6660	0.6487
SSE	0.0001	0.0001	0.0001	0.0002	0.0001
HYBRID	0.0005	0.0004	0.0004	0.0005	0.0007
ARE	0.2146	0.1890	0.1798	0.1885	0.2365
MPSD	0.4830	0.2913	0.2945	0.2309	0.5989
SAE	0.0402	0.0477	0.0479	0.0594	0.0389
SNE	3.5338	**3.1343**	3.4843	3.8650	4.0956

* Standard uncertainties of all constants are equal to 0.001, uncertainties of all errors equal to 0.0001 (0.95 level of confidence).

**Table 9:** Temkin isotherms constants with error analysis *.

	SSE	HYBRID	ARE	MPSD	SAE
M1_650_18N2_5CO2					
A_Te_	39.0636	57.0832	54.6758	77.1884	29.1599
b_Te_	1.3423	1.1523	1.1569	0.9819	1.4880
SSE	2.8067	4.0532	4.1562	9.0867	3.5988
HYBRID	8.7759	5.6327	5.7031	8.0215	17.4123
ARE	13.7056	12.9259	12.6175	14.4950	16.6299
MPSD	36.9524	21.5560	22.4965	17.3868	55.1802
SAE	7.5078	9.7911	9.7455	14.0762	6.7460
SNE	2.8401	**2.6330**	2.6437	3.6474	3.8753
M1_700_18N2_5CO2					
A_Te_	28.4636	43.6504	44.5742	60.3649	22.2786
b_Te_	1.2839	1.0520	1.0027	0.8499	1.4068
SSE	3.8159	5.6530	7.3710	13.3831	4.4069
HYBRID	14.1655	8.4241	8.9083	12.5809	24.7927
ARE	20.6212	17.6239	17.2926	19.1795	24.5633
MPSD	54.6068	29.1474	27.6047	22.7151	76.4514
SAE	9.0408	11.3395	12.4281	16.3965	8.4590
SNE	3.7894	**3.3144**	3.3451	3.8657	4.4115
M1_750_18N2_5CO2					
A_Te_	23.5056	38.4949	40.2880	55.2355	18.9426
b_Te_	0.9339	0.7216	0.6536	0.5417	1.0132
SSE	2.8629	4.4709	6.9353	11.4717	3.1416
HYBRID	17.3781	9.3338	10.5829	14.8482	27.9063
ARE	29.9893	23.5918	22.8205	24.8398	35.1854
MPSD	81.9577	39.2219	35.1320	28.9998	109.1639
SAE	7.9042	9.8863	11.5645	14.7386	7.5295
SNE	3.0117	**2.4248**	2.7388	3.5037	3.7847
M1_800_18N2_5CO2					
A_Te_	34.5219	49.6823	48.1275	65.4761	26.7146
b_Te_	0.4355	0.3735	0.3704	0.3178	0.4771
SSE	0.2672	0.3962	0.4432	0.9314	0.3337
HYBRID	2.9081	1.7778	1.8271	2.6060	5.6206
ARE	14.6073	13.3140	12.9974	14.8692	17.8082
MPSD	40.7303	22.6463	23.3231	17.9004	59.6674
SAE	2.3355	3.0471	3.1388	4.4590	2.1175
SNE	2.8309	**2.5522**	2.6255	3.5986	3.8332

* Standard uncertainties of all constants are equal to 0.001, uncertainties of all errors equal to 0.0001 (0.95 level of confidence).

**Table 10:** Dubinin-Raduskevich isotherms constants with error analysis *.

	SSE	HYBRID	ARE	MPSD	SAE
M1_650_18N2_5CO2					
q_mDR_	5.5232	5.2054	5.0767	4.7582	5.5182
A	0.2473	0.2063	0.2011	0.1677	0.2554
SSE	2.1219	2.7234	3.2053	5.5864	2.1805
HYBRID	4.3516	3.4223	3.5442	4.8396	4.7511
ARE	11.4669	10.0417	9.9046	10.7949	11.7751
MPSD	21.1864	16.1448	15.6698	13.5095	22.3152
SAE	7.1054	7.9096	8.2556	10.6944	7.0097
SNE	3.8667	**3.5106**	3.6214	4.5222	4.0275
M1_700_18N2_5CO2					
q_mDR_	5.1697	4.7281	4.4659	4.1497	5.2517
A	0.3304	0.2615	0.2370	0.2011	0.3613
SSE	2.0559	2.8318	4.0344	6.6112	2.2334
HYBRID	5.4779	4.1502	4.5250	6.2526	6.7690
ARE	14.7813	12.7453	12.2911	13.1042	15.7682
MPSD	26.9619	20.1217	17.9711	16.2888	29.9762
SAE	6.9716	8.0546	9.0181	11.2423	6.7422
SNE	3.5772	**3.2375**	3.4599	4.2982	3.9375
M1_750_18N2_5CO2					
q_mDR_	3.8257	3.3999	2.9995	2.8316	3.9027
A	0.4195	0.3204	0.2640	0.2305	0.4545
SSE	1.1112	1.6199	3.3040	4.4967	1.1763
HYBRID	4.7950	3.6034	4.6557	5.9481	5.6418
ARE	17.9809	15.5555	14.6740	15.5987	18.9140
MPSD	32.2971	24.5051	20.4933	19.3403	34.8375
SAE	5.1192	6.0827	7.6803	8.9868	4.9579
SNE	3.5006	**3.1688**	3.7362	4.3799	3.7618
M1_800_18N2_5CO2					
q_mDR_	1.7477	1.6461	1.5930	1.5100	1.7860
A	0.2630	0.2201	0.2114	0.1821	0.2902
SSE	0.1919	0.2497	0.3156	0.5060	0.2111
HYBRID	1.2611	0.9633	1.0207	1.3705	1.6942
ARE	11.4858	9.7739	9.5397	10.2021	12.7985
MPSD	21.0326	15.4396	14.5570	12.6304	24.6295
SAE	2.1512	2.3824	2.5380	3.1757	2.1128
SNE	3.5524	**3.2027**	3.3617	4.1189	4.0825

* Standard uncertainties of all constants are equal to 0.001, uncertainties of all errors equal to 0.0001 (0.95 level of confidence).

**Table 11:** Jovanovich isotherms constants with error analysis *.

	SSE	HYBRID	ARE	MPSD	SAE
M1_650_18N2_5CO2					
q_mJ_	5.4194	5.1152	4.9911	4.7085	5.5615
b_J_	2.7264	3.2195	3.3465	4.0947	2.4963
SSE	1.0637	1.3458	1.5577	2.9139	1.1653
HYBRID	2.3201	1.9783	2.0365	2.7522	2.8337
ARE	8.3191	7.9734	7.9055	8.4722	8.7905
MPSD	14.8355	12.5896	12.3751	11.1368	16.4127
SAE	4.8879	5.7567	5.9556	7.8879	4.6544
SNE	3.6537	**3.5639**	3.6616	4.6135	3.9900
M1_700_18N2_5CO2					
q_mJ_	5.5240	5.0989	5.0263	4.5291	5.6536
b_J_	1.8445	2.1831	2.1968	2.8242	1.7340
SSE	0.4811	0.6391	0.6906	1.6744	0.5227
HYBRID	1.3887	1.1663	1.2057	1.7741	1.6363
ARE	7.4004	6.9871	6.9262	7.5419	7.7863
MPSD	13.4231	11.3087	11.5029	9.7713	14.5339
SAE	3.3028	3.9509	3.9017	5.8755	3.1857
SNE	3.5062	3.3870	**3.4371**	4.6409	3.7767
M1_750_18N2_5CO2					
q_mJ_	4.7417	4.2422	4.0974	3.5891	4.9610
b_J_	1.2223	1.4701	1.5271	1.9628	1.1299
SSE	0.1309	0.1836	0.2328	0.5777	0.1427
HYBRID	0.6375	0.5265	0.5583	0.8762	0.7636
ARE	6.5500	6.0931	6.0251	6.6270	6.9888
MPSD	12.0953	10.1376	10.0994	8.5884	13.1436
SAE	1.7261	2.1118	2.1844	3.3855	1.6681
SNE	3.3215	**3.1857**	3.3159	4.6017	3.6112
M1_800_18N2_5CO2					
q_mJ_	1.7319	1.6367	1.5777	1.5142	1.7590
b_J_	2.5424	2.9612	3.1666	3.6542	2.3998
SSE	0.0793	0.1014	0.1336	0.2167	0.0848
HYBRID	0.5257	0.4380	0.4682	0.6189	0.6230
ARE	7.4001	6.9067	6.7912	7.0816	7.7519
MPSD	12.5888	10.3032	9.7884	8.8643	13.7982
SAE	1.3636	1.5771	1.6984	2.1198	1.3093
SNE	3.7200	**3.5526**	3.7545	4.5494	4.0090

* Standard uncertainties of all constants are equal to 0.001, uncertainties of all errors equal to 0.0001 (0.95 level of confidence).

**Table 12:** Radke-Prausnitz isotherms constants with error analysis *.

	SSE	HYBRID	ARE	MPSD	SAE
M1_650_18N2_5CO2					
q_mRP_	6.0801	6.0346	6.0345	6.0045	6.1359
b_RP_	7.1453	7.7204	7.7204	8.2684	6.5972
n_RP_	0.6237	0.6114	0.6107	0.5990	0.6353
SSE	0.0018	0.0027	0.0029	0.0059	0.0027
HYBRID	0.0057	0.0038	0.0039	0.0055	0.0128
ARE	0.3647	0.3350	0.3324	0.3670	0.4673
MPSD	0.9164	0.5601	0.5688	0.4408	1.4428
SAE	0.1993	0.2577	0.2611	0.3621	0.1796
SNE	2.7259	**2.5733**	2.6215	3.5170	3.9580
M1_700_18N2_5CO2					
q_mRP_	5.7020	5.6944	5.6881	5.6828	5.7173
b_RP_	5.5569	5.5968	5.6404	5.6899	5.4427
n_RP_	0.5016	0.5008	0.4998	0.4981	0.5042
SSE	0.0002	0.0001	0.0002	0.0002	0.0001
HYBRID	0.0002	0.0002	0.0002	0.0002	0.0003
ARE	0.0753	0.0656	0.0619	0.0651	0.0900
MPSD	0.1246	0.1044	0.0929	0.0871	0.2103
SAE	0.0511	0.0470	0.0491	0.0579	0.0456
SNE	2.9617	**2.5525**	2.7331	3.5854	3.8452
M1_750_18N2_5CO2					
q_mRP_	4.0263	3.6978	3.7420	4.1119	4.0787
b_RP_	5.8348	13.9867	11.7225	5.0523	5.3481
n_RP_	0.3585	0.3127	0.3203	0.3718	0.3647
SSE	0.0001	0.0014	0.0009	0.0002	0.0001
HYBRID	0.0003	0.0058	0.0048	0.0004	0.0003
ARE	0.1371	0.5852	0.5081	0.1379	0.1231
MPSD	0.3185	1.3273	1.2821	0.1701	0.2063
SAE	0.0444	0.1795	0.1349	0.0708	0.0473
SNE	1.8140	4.0187	3.0246	**0.2076**	0.4890
M1_800_18N2_5CO2					
q_mRP_	2.0027	1.8197	2.0092	2.0170	1.9999
b_RP_	5.0158	12.7788	4.8565	4.7483	5.0536
n_RP_	0.6512	0.5146	0.6605	0.6660	0.6503
SSE	0.0001	0.0133	0.0001	0.0002	0.0001
HYBRID	0.0005	0.0746	0.0005	0.0005	0.0006
ARE	0.2146	2.6605	0.1799	0.1885	0.2209
MPSD	0.4830	4.9792	0.2943	0.2309	0.5463
SAE	0.0402	0.5515	0.0480	0.0594	0.0388
SNE	0.2633	5.0000	**0.2306**	0.2450	0.2773

* Standard uncertainties of all constants are equal to 0.001, uncertainties of all errors equal to 0.0001 (0.95 level of confidence).

## Data Availability

Not applicable.
